# Analysis of 386 alternative medicinal products implicated in liver injury reveal clinically relevant associations with potentially hepatotoxic botanicals, pharmaceutical adulteration, heavy metal contamination, and undisclosed animal content

**DOI:** 10.3389/fgstr.2026.1784785

**Published:** 2026-03-11

**Authors:** Cyriac Abby Philips, Tharun Tom Oommen, Arif Hussain Theruvath, Aryalakshmi Sreemohan, Ambily Baby, Rizwan Ahamed, Ajit Tharakan, Philip Augustine

**Affiliations:** 1 Department of Clinical and Translational Hepatology, The Liver Institute, Center of Excellence in Gastrointestinal Sciences, Rajagiri Hospital, Aluva, Kerala, India; 2 Clinical Research Division, The Liver Institute, Center of Excellence in Gastrointestinal Sciences, Rajagiri Hospital, Aluva, Kerala, India; 3 Division of Complementary and Alternative Medicine and The Liver, The Liver Institute, Center of Excellence in Gastrointestinal Sciences, Rajagiri Hospital, Aluva, Kerala, India; 4 Department of Gastroenterology and Advanced GI Endoscopy, Center of Excellence in Gastrointestinal Sciences, Rajagiri Hospital, Aluva, Kerala, India

**Keywords:** ACLF, ALF, Ayurveda, AYUSH, herbal and dietary supplements, homeopathy

## Abstract

**Background:**

Complementary and alternative medicine (CAM)-related hepatotoxicity is a growing global concern. We utilized multi-modal analysis to characterize CAM product safety and identify predictors of severe liver injury.

**Methods:**

This retrospective study analyzed 386 CAM products from 91 consecutive patients (mean 4.2 products/patient) presenting with CAM-related adverse events at a tertiary center in South India (2021–2023). Product-level analyses characterize the CAM supply chain while patient-level analyses inform clinical outcome associations. Investigations included ingredient documentation, heavy metal quantification, and GC-MS compound profiling.

**Results:**

The mean patient age was 48.2 years (75.8% male). ACLF occurred in 39.6% of all patients (36/91) and 41.9% of those with hepatic adverse events (36/86), with associated mortality of 38.9% (14/36) compared to 10.9% (6/55) in non-ACLF presentations (OR 5.20, P = 0.004). Heavy metals exceeded WHO limits in many products: mercury (34%), cadmium (25%), arsenic (21%), and lead (14%). Cadmium exposure exceeding WHO limits showed a strong association with ACLF (75.9% vs 22.6%, P<0.001, FDR q<0.001). The association with mortality did not reach statistical significance after correction for multiple comparisons (34.5% vs 16.1%, uncorrected P = 0.061, FDR q=0.24). Undeclared pharmaceutical adulteration (at least one adulterant found in 27.7% of products; exposure to at least one adulterated product in 46.2% of patients) and animal-derived content (31.3%) were prevalent. Notably, unlabeled product consumption significantly predicted mortality (P = 0.025).

**Conclusion:**

CAM-associated liver injury frequently manifests as ACLF with high mortality, driven by pervasive heavy metal contamination and adulteration. Unlabeled product exposure is a strong mortality predictor, highlighting the urgent need for mandatory product surveillance.

## Introduction

The global utilization of complementary and alternative medicine (CAM), encompassing herbal and dietary supplements (HDS), traditional medicine systems such as Ayurveda, Siddha, and Traditional Chinese Medicine (TCM), has witnessed exponential growth over the past two decades. According to a recent analysis of the National Health and Nutrition Examination Survey (NHANES), an estimated 15.6 million adults in the United States consumed at least one potentially hepatotoxic botanical product within a 30-day period, a number comparable to consumers of prescription hepatotoxic medications such as simvastatin and non-steroidal anti-inflammatory drugs (1). This widespread use, driven by perceptions of natural safety and ‘promoted’ efficacy, has been accompanied by a concerning increase in CAM-related adverse events, particularly hepatotoxicity. Data from the US Drug-Induced Liver Injury Network (DILIN) demonstrate that HDS account for approximately 16–20% of drug-induced liver injury (DILI) cases, with herbal products increasingly recognized as important causes of acute liver failure requiring transplantation (2). A nationwide prospective study from Iceland revealed DILI prevalence of 8% among patients with elevated liver enzymes, with herbal supplements representing a significant causative category and associated with emergency liver transplantation (3). The Latin American DILI Network’s 10-year experience documented that HDS accounted for 9% of all DILI cases, with these products associated with the highest mortality rates compared to conventional pharmaceuticals (4).

The burden of CAM-related hepatotoxicity is particularly substantial in South and Southeast Asia, where traditional medicine systems are deeply embedded in healthcare practice. The Asia Pacific Association for the Study of the Liver (APASL) consensus guidelines on DILI underscore that the incidence of DILI is likely higher in Asia than other regions worldwide, predominantly due to hepatotoxicity from antituberculosis (ATT) treatment and the ubiquitous use of traditional and complementary medicines (5). Analysis of the APASL ACLF Research Consortium (AARC) database comprising 3,132 patients with ACLF demonstrated that while alcohol (48.2%) and hepatitis B reactivation (12.7%) were the predominant ACLF precipitants, drugs precipitated ACLF in 10.5% of cases; among drug-induced ACLF cases, CAMs constituted 71.7% (6).

In India, the Ministry of AYUSH (Ayurveda, Yoga, Unani, Siddha, and Homeopathy) regulates a vast traditional medicine industry; however, regulatory oversight remains limited, particularly for unlabeled products obtained directly from traditional practitioners. A prospective cohort from South India comprising 133 patients with DILI reported that ATT (37.5%), neuropsychiatric drugs (16.5%), and CAM products (10.5%) were the predominant etiologies, with 13.5% mortality; MELD score >28, INR >1.97, and bilirubin >15.6 mg/dL independently predicted mortality (7). Recent multi-center case series from India have characterized the hepatotoxicity profiles of specific traditional medicine products. The first and largest Indian case series on ashwagandha-induced liver injury from multiple centers reported 100% mortality among those developing acute-on-chronic liver failure (ACLF), highlighting the heightened vulnerability of those with pre-existing hepatic compromise (8). A series of patients with homeopathic remedy-induced severe DILI demonstrated that toxicological analysis of the implicated formulations revealed industrial solvents, corticosteroids, antibiotics, sedatives, synthetic opioids, heavy metals, and toxic phytocompounds even in purportedly ultra-dilute preparations, with 44.4% mortality predominantly among those with underlying chronic liver disease (9). During the COVID-19 pandemic, widespread self-medication with *Tinospora cordifolia* (Giloy) – promoted as an ‘immune booster’ – led to documented hepatotoxicity, particularly among individuals with unrecognized pre-existing liver disease, those consuming excessive doses, or those using unverified preparations, raising concerns that this herb may trigger or unmask autoimmune hepatitis in susceptible individuals (10). In a large retrospective study of 1,666 patients with cirrhosis, 68% reported CAM use at some point; among patients developing CAM-related decompensation, 35.7% progressed to ACLF, with 53% overall mortality and unlabeled multi-herbal Ayurvedic formulations as the most common implicated agents (11). Furthermore, HDS use among patients with liver disease is highly prevalent yet frequently underreported, with structured interviews revealing 61% consumption rates compared to only 8% detected through standard clinical history (12).

The safety concerns surrounding CAM products extend beyond their botanical constituents to include widespread contamination and adulteration. A landmark study employing combined DNA sequencing, toxicological screening, and heavy metal analysis revealed that 92% of TCM products examined had some form of contamination or substitution, with 50% containing undeclared pharmaceutical agents including warfarin, dexamethasone, and diclofenac, and heavy metal levels exceeding acceptable limits by more than tenfold (13). A systematic review of herbal medicinal product contamination documented severe adverse effects including multi-organ failure, hepatorenal syndrome, and death, with traditional Indian and Chinese remedies most commonly implicated (14). Furthermore, investigation by the DILIN consortium demonstrated that chemical analysis of HDS products led to increased confidence in DILI attribution in 37% of cases, highlighting the critical role of comprehensive product characterization in causality assessment (15).

Despite the growing recognition of CAM-related hepatotoxicity, significant knowledge gaps persist. First, comprehensive multi-modal product analysis integrating ingredient documentation, heavy metal quantification, and gas chromatography-mass spectrometry (GC-MS) compound profiling has rarely been applied systematically to products implicated in adverse events. Second, the extent of pharmaceutical adulteration in South-Asian medicine products (predominantly Indian subcontinent) and its correlation with clinical outcomes remains inadequately characterized. Third, the contribution of undisclosed animal-derived ingredients, relevant to vegetarian consumers, religious dietary restrictions, and endangered species conservation, has not been systematically evaluated. Fourth, predictors of severe outcomes including ACLF and mortality in CAM-related hepatotoxicity require further elucidation. Fifth, the safety profile of unlabeled products obtained from informal markets and traditional practitioners, which constitute a substantial proportion of CAM consumption in developing countries, remains poorly understood.

In this context, the primary aim of this study was to perform a comprehensive, multi-dimensional analysis of CAM products associated with adverse events in patients presenting to a tertiary hepatology center in South India, integrating systematic ingredient documentation, heavy metal analysis, and GC-MS compound profiling to characterize the botanical, chemical, and pharmaceutical composition of implicated products. The secondary aims were: (1) to determine the prevalence and predictors of mortality and ACLF in CAM-related hepatotoxicity; (2) to quantify the extent of pharmaceutical adulteration, heavy metal contamination, and undisclosed animal-derived content across different traditional medicine systems; (3) to identify associations between specific ingredients, compounds, and clinical outcomes; and (4) to evaluate the safety profile of unlabeled versus labeled products.

## Patients and methods

This retrospective observational study included consecutive patients who presented with adverse events attributed to CAM product consumption (after reasonable exclusion of other competing causes through clinical, serological, and imaging evaluation) from May 2021 to December 2023 to a tertiary care hepatology center in South India. Formal causality assessment tools such as RUCAM were not applied, as these instruments were designed for single-drug exposures and have recognized limitations in multi-product CAM scenarios and where product composition is frequently unknown or adulterated. Attribution was based on temporal association, exclusion of competing etiologies, and expert hepatologist adjudication, consistent with the pragmatic approach recommended for complex HILI presentations. The study was conducted with systematic collection of both clinical data and physical CAM product samples for comprehensive laboratory analysis. Inclusion criteria comprised: (1) patients of any age presenting with documented adverse events temporally associated with CAM product use; (2) availability of retrievable CAM product samples for laboratory analysis; and (3) adequate clinical documentation for outcome assessment. Exclusion criteria included: (1) concurrent use of known hepatotoxic prescription medications that could confound causality assessment; (2) incomplete clinical documentation precluding outcome determination and (3) patients with hepatic or extrahepatic malignancy. CAM products were obtained directly from patients or their family members or were handed over by primary treating physicians. All products underwent systematic classification according to traditional medical system of origin, ingredient documentation through label analysis and expert consultation, and comprehensive laboratory analysis. Product labeling status was documented as either labeled (products with manufacturer identification, batch numbers, ingredient lists, and manufacturing/expiry dates) or unlabeled (products lacking formal identification). For exposure characterization, product-level statistics (proportion of products containing a given contaminant) are reported to describe the quality and safety profile of the CAM supply chain. For clinical outcome analyses, patient-level exposure was defined as exposure to at least one product containing the contaminant of interest, recognizing that products from the same patient are non-independent observations. This approach ensures that patients consuming multiple products do not disproportionately influence outcome associations. Both product-level and patient-level exposure data are reported in the results to provide complementary perspectives on contamination prevalence. Heavy metal concentrations for 14 elements of toxicological concern where possible, including iron, mercury, lead, arsenic, cadmium, copper, zinc, tin, silver, gold, chromium, manganese, nickel, and antimony were determined using an inductively coupled plasma-atomic emission spectrometer (IRIS Intrepid II XSP Duo; Thermo Fisher Scientific, Waltham, MA, USA) using chemical standards and reagents according to the United States Environmental Protection Agency standard methods 5021A, 8015, 8021, and 8260. Heavy metal concentrations were compared against World Health Organization (WHO) guidelines for heavy metals in herbal medicines. These limits (lead ≤10 ppm, arsenic ≤3 ppm, mercury ≤1 ppm, cadmium ≤0.3 ppm) were established for finished herbal products intended for regular consumption and represent concentration thresholds in the product rather than daily intake limits. Other results were interpreted against established safety thresholds where available. All retrieved products underwent exhaustive triple-quadruple gas chromatography coupled with tandem mass spectrometry (GC–MS/MS; Thermo Fisher Scientific, Waltham, MA, USA) analysis for identification of volatile and semi-volatile organic compounds, pharmaceutical adulterants, and chemical markers of ingredient composition and impurities. Sample preparation involved solvent extraction with appropriate solvents based on product matrix. Analysis was performed using electron ionization with compound identification performed using NIST Mass Spectral Library (NIST 17) matching with a minimum match factor threshold of ≥700 (out of 1000) for positive identification, corresponding to a ‘good’ or ‘excellent’ match per NIST guidelines. Retention index comparison against published values was used as secondary confirmation where available. Semi-quantitative peak area data were generated but not used for dose-response analysis given the complexity of matrix effects in multi-ingredient botanical products and the absence of validated internal standards for all detected compounds. The analysis was therefore treated as qualitative (presence/absence) for statistical purposes. GC-MS/MS was selected as the primary analytical platform for its broad-spectrum untargeted screening capability across diverse chemical matrices, extensive spectral library support (NIST 17, >300,000 reference spectra), and established application in traditional medicine pharmacovigilance.

### Statistical analysis

Continuous variables were expressed as mean ± standard deviation (SD) for normally distributed data and as median with interquartile range (IQR) for non-normally distributed data. Normality was assessed using the Shapiro-Wilk test. Categorical variables were expressed as frequencies and percentages with 95% confidence intervals where appropriate. Comparative analyses between groups were performed using Chi-square tests for categorical variables with expected cell frequencies ≥5, and Fisher’s exact tests when expected frequencies were <5. Continuous variables were compared using independent samples t-tests for normally distributed data or Mann-Whitney U tests for non-normally distributed data. All tests were two-tailed with α=0.05. Binary logistic regression analysis was performed to identify independent predictors of clinical outcomes. Variables demonstrating significance at p<0.10 in univariate analysis or with established clinical importance were included in multivariate models. Results were expressed as odds ratios (OR) with 95% confidence intervals (CI). Associations between specific ingredients, GC-MS compounds, heavy metals, and clinical outcomes were evaluated using Chi-square tests with Yates’ continuity correction or Fisher’s exact tests as appropriate. To account for the non-independence of multiple products consumed by the same patient, all clinical outcome analyses (ACLF and mortality associations) were conducted at the patient level, with exposure defined as consumption of at least one product containing the contaminant or compound of interest. Product-level statistics are reported separately to characterize the CAM supply chain. For the large number of ingredient and compound comparisons, only items present in ≥5 products were included in formal statistical testing to ensure adequate statistical power, with findings interpreted in the context of biological plausibility and effect size magnitude. To address the issue of multiple comparisons in exploratory analyses, the Benjamini-Hochberg procedure was applied to control the false discovery rate (FDR) at 0.05. For pre-specified primary analyses (ACLF-mortality association, unlabeled product consumption-mortality association), uncorrected P-values <0.05 were considered statistically significant. For exploratory secondary analyses involving multiple compounds, ingredients, or heavy metals, FDR-adjusted q-values are reported alongside uncorrected P-values. Associations with q<0.05 were considered statistically significant after correction. All statistical analyses were performed using Python 3.11 with scipy.stats (version 1.11.2) and statsmodels (version 0.14.0) libraries.

## Results

### Patient characteristics and clinical outcomes

#### Demographic characteristics

**Figure 1:**
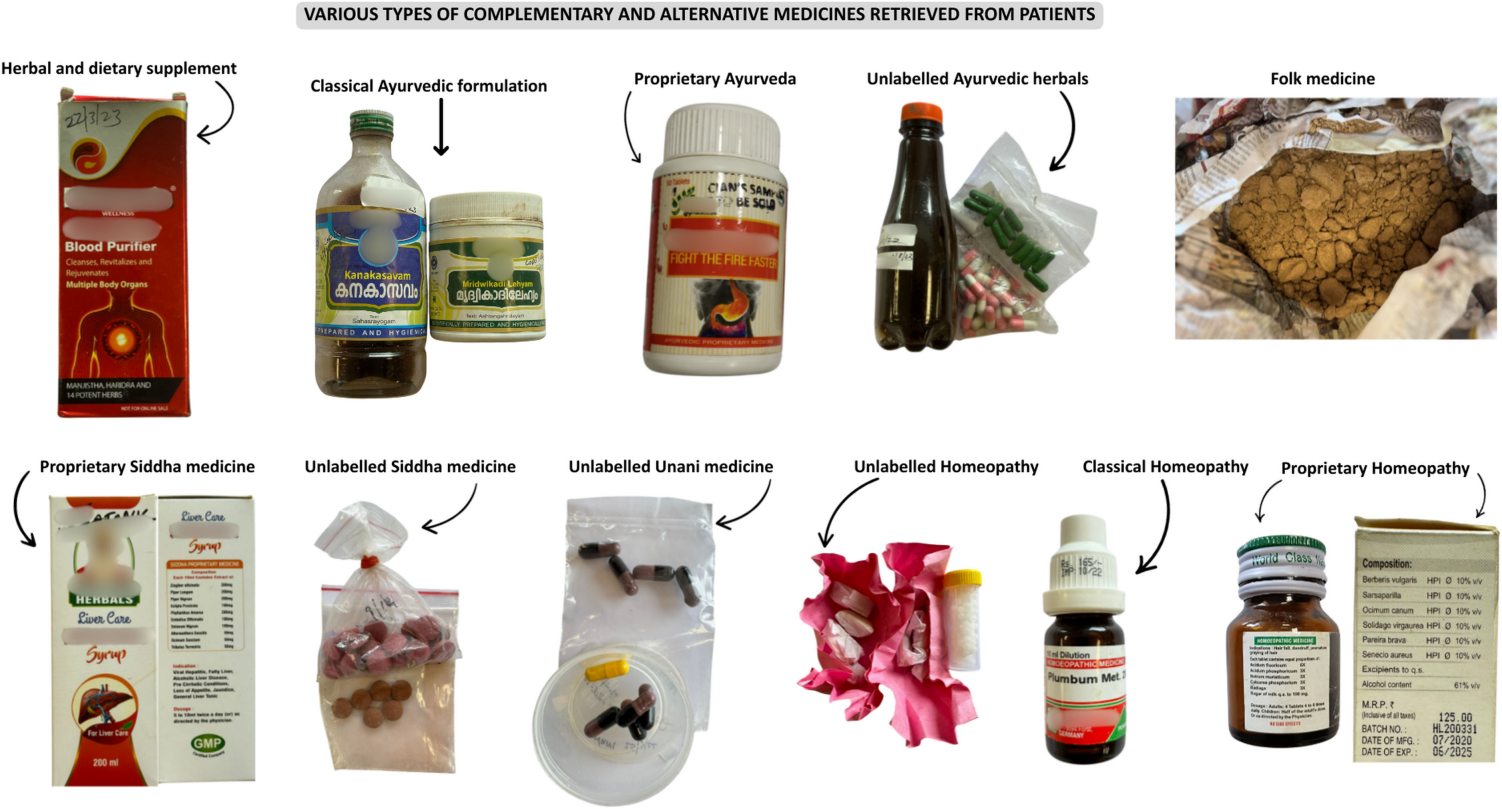
Representative samples of various types of complementary and alternative medicines retrieved for analysis from patients.

The study cohort comprised 91 patients with a mean age of 48.2 ± 14.9 years (median 49, IQR 38-59, range 14–81 years). The majority were male (n=69, 75.8%) with a male-to-female ratio of 3.1:1. There was no significant difference in age between survivors and non-survivors (48.1 ± 15.1 vs. 48.6 ± 14.5 years; Mann-Whitney U test p=0.981), nor between patients with and without ACLF (47.8 ± 14.4 vs. 48.5 ± 15.3 years; p=0.948). Sex distribution was similar between outcome groups (male mortality 21.7% vs. female mortality 22.7%). Patients consumed a mean of 4.2 ± 3.7 CAM products (median 3, IQR 2-6, range 1-18), with a total of 386 distinct (**Figure 1**) CAM product samples retrieved for analysis. The number of products consumed did not differ significantly between survivors and non-survivors (4.2 ± 3.8 vs. 4.5 ± 3.6; Mann-Whitney U p=0.428) or between patients with and without ACLF (4.8 ± 4.0 vs. 3.9 ± 3.5; p=0.226).

#### Type and pattern of adverse events

**Figure 2:**
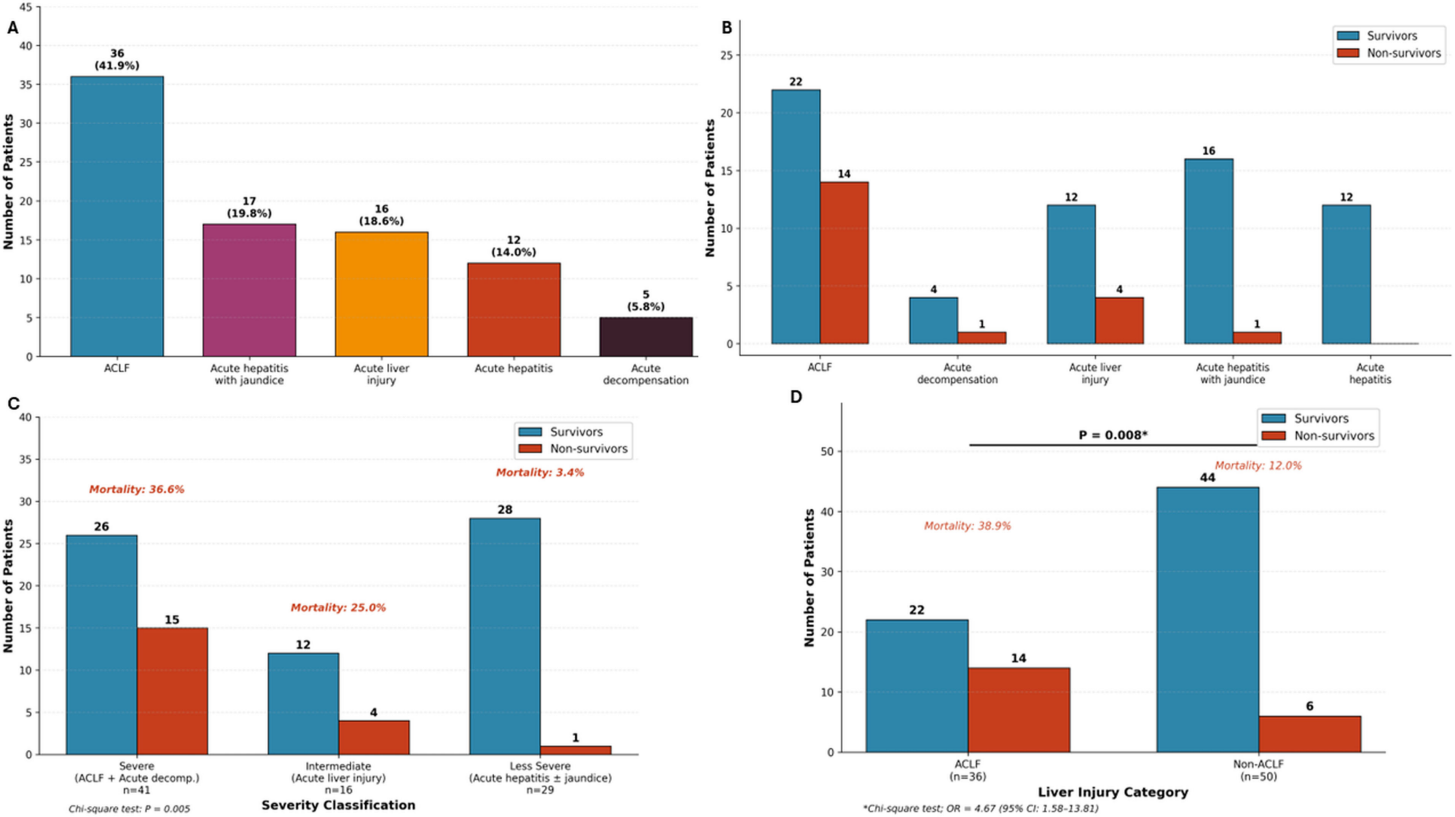
Clinical presentations and outcomes of CAM-related hepatotoxicity. **(A)** Distribution of liver injury patterns among 86 patients with hepatic adverse events. Acute-on-chronic liver failure (ACLF) was the most common presentation (36 patients, 41.9%), followed by acute hepatitis with jaundice (17 patients, 19.8%), acute liver injury (16 patients, 18.6%), acute hepatitis (12 patients, 14.0%), and acute decompensation (5 patients, 5.8%). **(B)** Survival outcomes stratified by liver injury pattern. Non-survivors (red bars) were most frequent in the ACLF group (14/36, 38.9%), followed by acute liver injury (4/16, 25.0%), acute decompensation (1/5, 20.0%), and acute hepatitis with jaundice (1/17, 5.9%). No deaths occurred among patients with acute hepatitis (0/12). **(C)** Mortality rates by severity classification. Patients were grouped into severe (ACLF plus acute decompensation, n=41), intermediate (acute liver injury, n=16), and less severe (acute hepatitis with or without jaundice, n=29) categories. Mortality rates demonstrated a significant gradient across severity groups: 36.6% (15/41) for severe, 25.0% (4/16) for intermediate, and 3.4% (1/29) for less severe presentations (Chi-square test for trend, P = 0.005). **(D)** Comparison of mortality between ACLF and non-ACLF presentations. Patients with ACLF had significantly higher mortality (38.9%, 14/36) compared to non-ACLF patients (10.9%, 6/55); odds ratio 5.20 (95% CI: 1.76–15.31), P = 0.004 by Fisher’s exact test.

Hepatic adverse events were documented in 82 patients (90.1%), while 9 patients (9.9%) experienced extrahepatic manifestations either alone or in combination with hepatic injury. As four patients had both hepatic and extrahepatic presentations, the total number of patients with any hepatic involvement was 86. Extrahepatic manifestations included dermatological reactions (n=3, 3.3%), cardiac toxicity (n=1, 1.1%), and combined anemia with gastrointestinal symptoms (n=1, 1.1%). Four patients (4.4%) experienced both hepatic and extrahepatic adverse events. Among patients with hepatic involvement, ACLF was the most common presentation, affecting 36 patients (39.6%) with a mortality rate of 38.9% (14/36). Other presentations included acute hepatitis with jaundice (n=17, 18.7%; mortality 5.9%), acute liver injury (n=16, 17.6%; mortality 25.0%), acute hepatitis without prominent jaundice (n=12, 13.2%; mortality 0%), and acute decompensation of underlying chronic liver disease (n=5, 5.5%; mortality 20.0%). The striking difference in mortality across liver injury patterns was statistically significant (Chi-square p<0.001) (**Figures 2A, B**).

#### Clinical outcomes and predictors

At last follow-up, 65 patients (71.4%) were alive, 20 patients (22.0%) had died, and one patient (1.1%) underwent liver transplantation, yielding an overall mortality rate of 22.0% (20/91). Five patients (5.5%) had incomplete outcome documentation and were classified as non-ACLF presentations with extrahepatic adverse events; these patients were included in all analyses with unknown outcomes treated conservatively as non-deaths. ACLF was strongly associated with mortality. Among ACLF patients, 38.9% (14/36) died compared to 10.9% (6/55) among non-ACLF patients. The crude odds ratio was 5.20 (95% CI: 1.76-15.31; Fisher’s exact p=0.0036). This association remained significant in multivariate analysis adjusting for age and sex (adjusted OR 4.67, 95% CI 1.52-14.35, p=0.008), establishing ACLF as the strongest independent predictor of mortality in this cohort (**Figures 2C, D**). In sensitivity analysis excluding the five patients with incomplete outcome documentation (all non-ACLF, extrahepatic presentations), the mortality rate was 23.3% (20/86), and the ACLF-mortality association was unchanged (OR 4.67, 95% CI: 1.58–13.81, P = 0.005), confirming robustness of the primary finding.

### Detailed analysis of CAM product types

**Figure 3:**
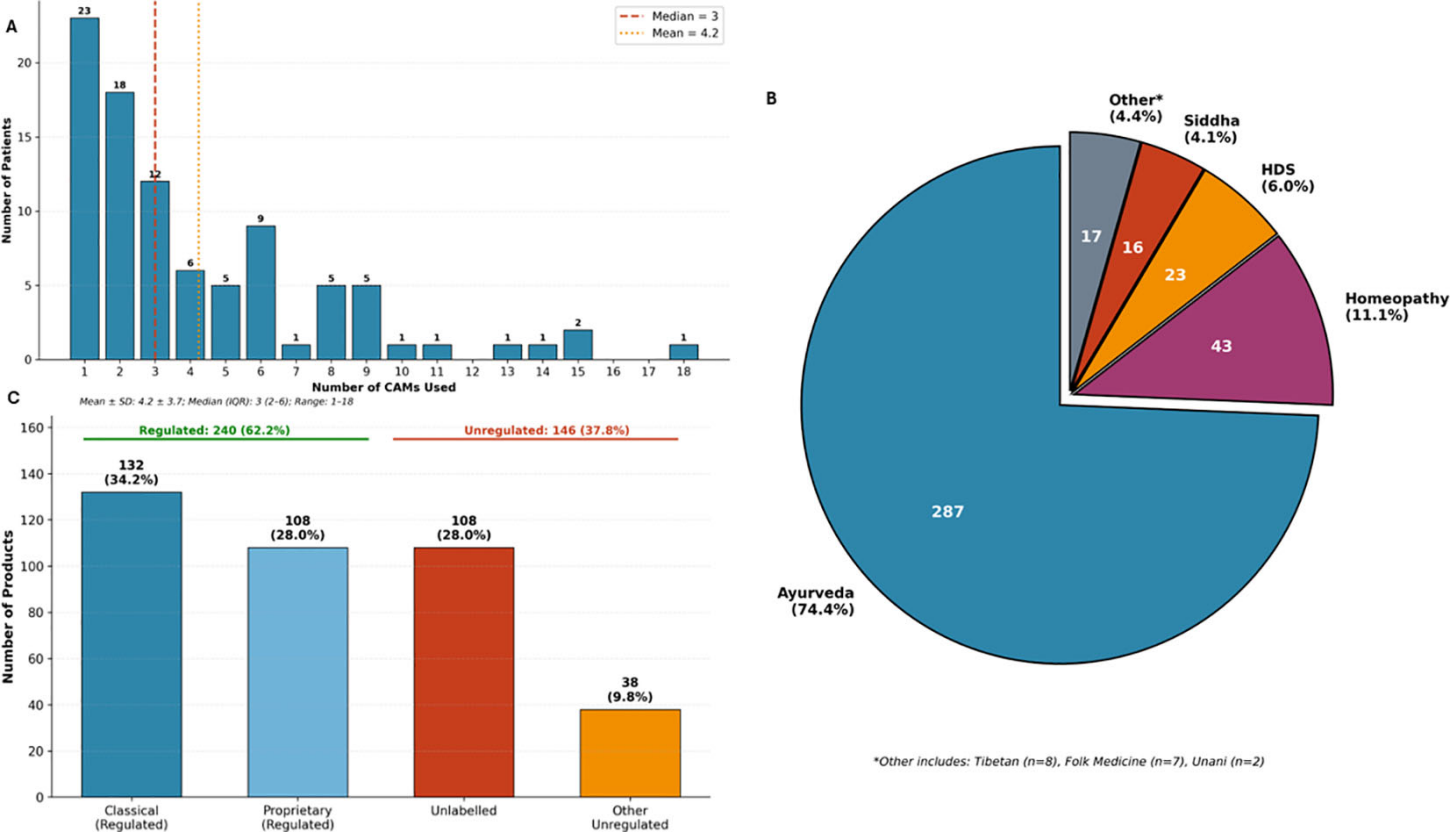
CAM product characteristics and classification. **(A)** Distribution of the number of CAM products consumed per patient (N = 91). Patients used a mean of 4.2 ± 3.7 products (median 3, IQR 2–6, range 1–18). Dashed lines indicate median (red) and mean (yellow) values. **(B)** Distribution of 386 CAM products by traditional medical system. Ayurveda predominated (n=287, 74.4%), followed by Homeopathy (n=43, 11.1%), Herbal and Dietary Supplements (HDS; n=23, 6.0%), Siddha (n=16, 4.1%), and other systems including Tibetan (n=8), Folk Medicine (n=7), and Unani (n=2), collectively comprising 4.4%. **(C)** Product distribution by regulatory status. Regulated products comprised 62.2% (n=240), including Classical formulations (n=132, 34.2%) and Proprietary preparations (n=108, 28.0%). Unregulated products comprised 37.8% (n=146), including Unlabeled preparations (n=108, 28.0%) and other unregulated categories (n=38, 9.8%).

Among 386 CAM products implicated in adverse events across 91 patients, Ayurvedic preparations predominated, comprising nearly three-quarters of the cohort (287 products, 74.4%), followed by Homeopathy (43, 11.1%), herbal/dietary supplements (23, 6.0%), Siddha (16, 4.1%), Tibetan medicine (8, 2.1%), folk medicine (7, 1.8%), and Unani (2, 0.5%). By regulatory status, 62.2% (240/386) were regulated products – including classical formulations (132, 34.2%) and proprietary preparations (108, 28.0%), while 37.8% (146/386) were unregulated, predominantly unlabeled products obtained from both registered Ayurveda, Homeopathy, Unani and Siddha as well as folk/traditional practitioners (108, 28.0%) (**Figure 3**).

**Figure 4:**
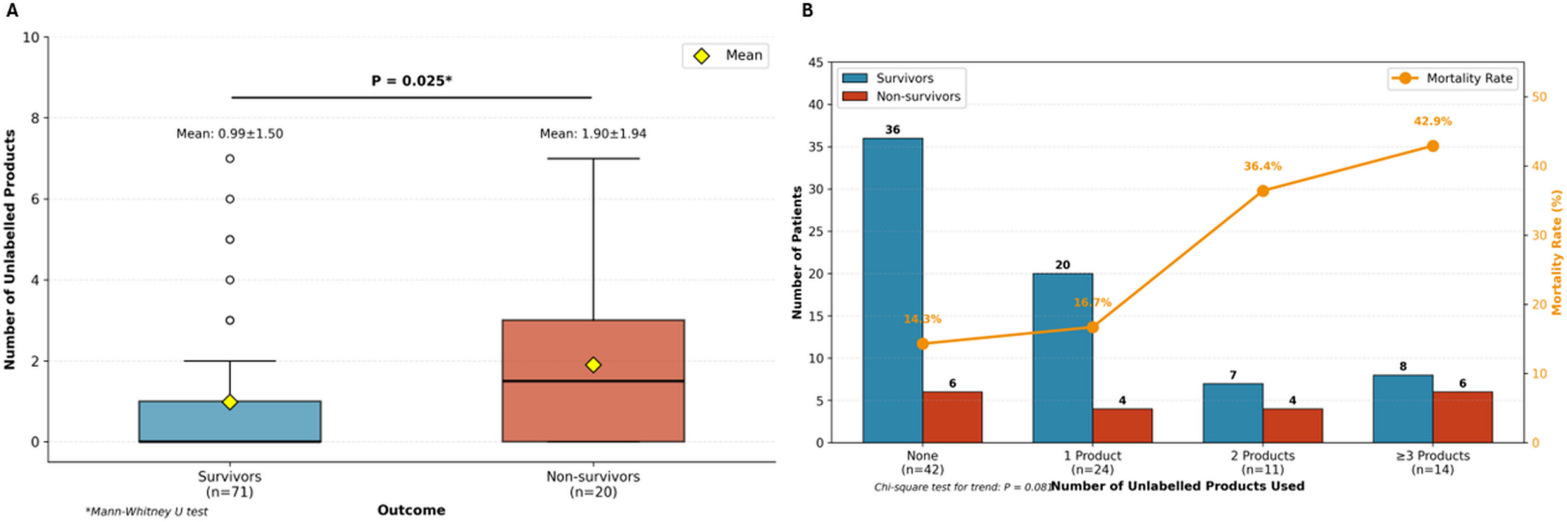
Association between unlabeled CAM product consumption and mortality. **(A)** Box plot comparing the number of unlabeled CAM products consumed between survivors (n=71) and non-survivors (n=20). Non-survivors consumed significantly more unlabeled products than survivors (mean 1.90 ± 1.94 vs 0.99 ± 1.50; Mann-Whitney U test, P = 0.025). Diamonds indicate mean values; horizontal lines within boxes indicate medians. **(B)** Dose-response relationship between unlabeled product consumption and mortality. Mortality rates increased progressively from 14.3% in patients using no unlabeled products (n=42), to 16.7% with one product (n=24), 36.4% with two products (n=11), and 42.9% with three or more products (n=14). Blue bars represent survivors; red bars represent non-survivors; orange line depicts mortality rate (right y-axis). Chi-square test for trend, P = 0.08.

A critical finding was the significant association between unlabeled product consumption and mortality (**Figure 4**): non-survivors consumed nearly twice as many unlabeled products as survivors (1.90 ± 1.94 vs 0.99 ± 1.50, P = 0.025), with a dose-response relationship demonstrating mortality rates escalating from 14.3% among patients using no unlabeled products to 42.9% among those consuming three or more (P for trend = 0.081) Notably, no specific traditional medicine system was independently associated with mortality, suggesting that the regulatory status and traceability of products, rather than the medical tradition from which they originate, may be the more critical determinant of patient safety outcomes.

### Ingredient analysis in CAM products

#### Overview of disclosed ingredient composition

**Figure 5:**
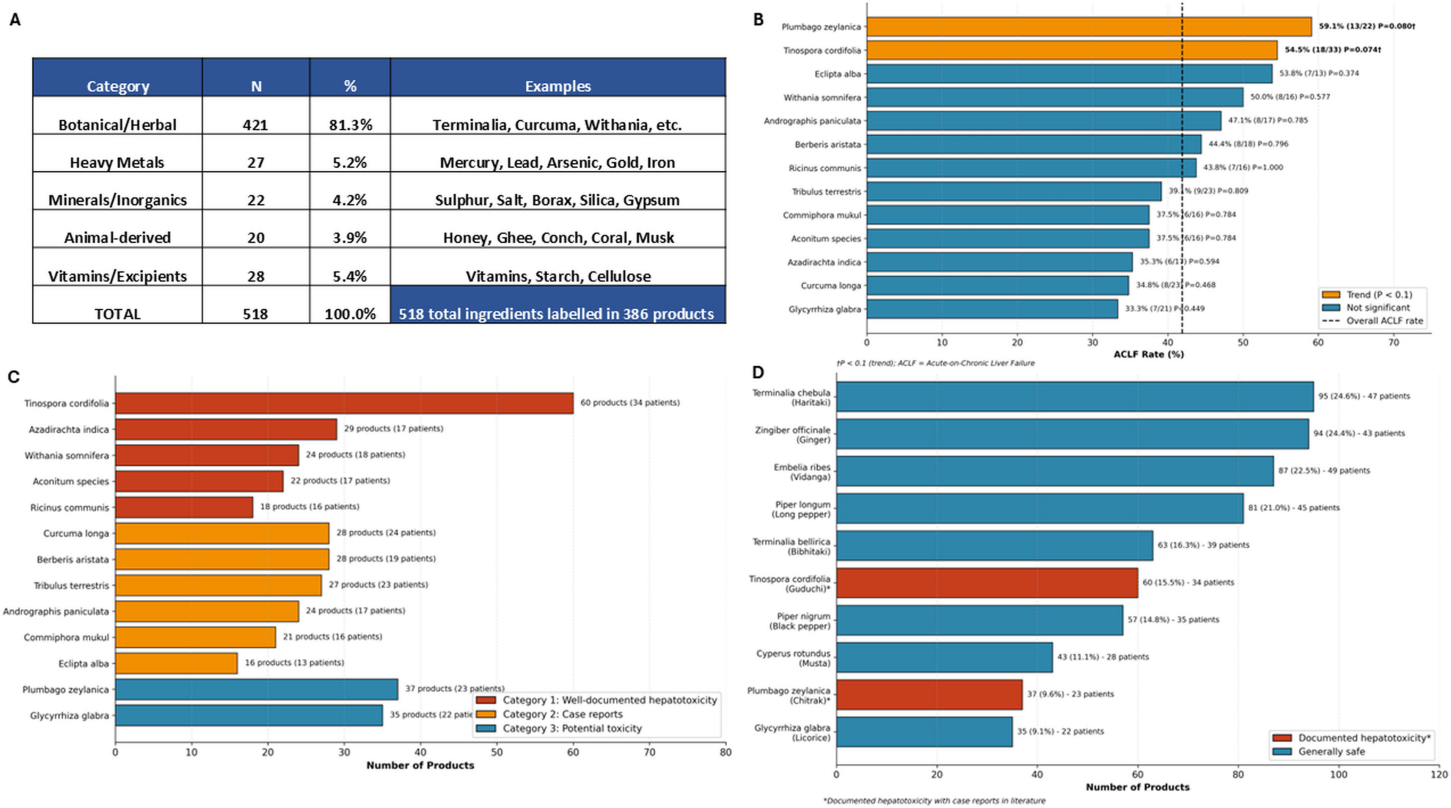
Ingredient analysis and hepatotoxic botanical identification. **(A)** Distribution of 518 unique ingredients identified across 386 CAM products by category: Botanical/Herbal (n=421, 81.3%), Vitamins/Excipients (n=28, 5.4%), Heavy Metals (n=27, 5.2%), Minerals/Inorganics (n=22, 4.2%), and Animal-derived (n=20, 3.9%). **(B)** ACLF rates associated with exposure to hepatotoxic botanical ingredients. *Plumbago zeylanica* (59.1%, P = 0.080) and *Tinospora cordifolia* (54.5%, P = 0.074) showed trending associations with ACLF development. Dashed line indicates overall ACLF rate (41.9%); orange bars indicate trending associations (P<0.1); blue bars indicate non-significant associations. **(C)** Hepatotoxic botanicals categorized by evidence level: Category 1 (red) = well-documented hepatotoxicity (*Tinospora cordifolia*, *Azadirachta indica*, *Withania somnifera*, *Aconitum* species, *Ricinus communis*); Category 2 (orange) = case reports (*Curcuma longa*, *Berberis aristata*, *Tribulus terrestris*, *Andrographis paniculata*, *Commiphora mukul*, *Eclipta alba*); Category 3 (blue) = potential toxicity based on toxic compounds (*Plumbago zeylanica*, *Glycyrrhiza glabra*). **(D)** Top 10 most prevalent botanical ingredients with hepatotoxicity documentation status. Asterisks indicate botanicals with documented hepatotoxicity in published literature.

Systematic ingredient documentation across all 386 CAM products identified 518 unique ingredients, distributed across five major categories: botanical/herbal ingredients (n=421, 81.3%), vitamins and pharmaceutical excipients (n=28, 5.4%), heavy metals (n=27, 5.2%), minerals and inorganic compounds (n=22, 4.2%), and animal-derived components (n=20, 3.9%). The most frequently encountered botanical ingredients were: *Terminalia chebula* (haritaki) present in 95 products (24.6%), *Zingiber officinale* (ginger) in 94 products (24.4%), *Embelia ribes* (vidanga) in 87 products (22.5%), *Piper longum* (long pepper) in 81 products (21.0%), and *Terminalia bellirica* (bibhitaki) in 63 products (16.3%). The classical Triphala formulation components (*Terminalia chebula, Terminalia bellirica, and Emblica officinalis*) were present individually or in combination in over 40% of Ayurvedic products (**Figure 5A**).

#### Labeled (without warning) hepatotoxic botanical identification

Cross-referencing ingredients against the LiverTox database and published literature identified that 135 products (35.0%) contained at least one botanical ingredient with documented or potential hepatotoxicity. Products containing botanicals with any documented hepatotoxicity comprised 42% (162/386) of the cohort, with 25.6% (99/386) containing Category 1 (well-documented) hepatotoxins. At the patient level, 69.2% (63/91) were exposed to at least one documented hepatotoxic botanical, and 51.6% (47/91) to Category 1 hepatotoxins. The most prevalent botanicals with well-documented hepatotoxicity included *Tinospora cordifolia* (Giloy or Guduchi; 60 products, 15.5%), *Azadirachta indica* (Neem; 29 products, 7.5%), *Withania somnifera* (Ashwagandha; 24 products, 6.2%), *Aconitum* species (22 products, 5.7%), and *Ricinus communis* (Castor; 18 products, 4.7%). Additionally, botanicals containing potentially hepatotoxic compounds were identified, notably *Plumbago zeylanica* (Chitrak; 37 products) containing the quinoid toxin plumbagin, and *Glycyrrhiza glabra* (Licorice; 35 products) with dose-dependent glycyrrhizin toxicity. Clinical outcome analysis revealed trending associations between specific hepatotoxic botanicals and ACLF development. *Tinospora cordifolia* exposure demonstrated an ACLF rate of 54.5% compared to 34.0% in unexposed patients (uncorrected P = 0.050, FDR q=0.50), while *Plumbago zeylanica* exposure showed an ACLF rate of 59.1% versus 35.9% in unexposed patients (uncorrected P = 0.083, FDR q=0.50). Neither association remained statistically significant after correction for multiple comparisons across 518 tested ingredients (**Figures 5B-D**). These findings suggest that while hepatotoxic botanical exposure may influence disease severity progression to ACLF, the ultimate mortality outcome is likely determined by multifactorial product, host and environmental variables rather than specific botanical constituents alone.

#### Labeled and detected animal derived ingredient identification

**Figure 6:**
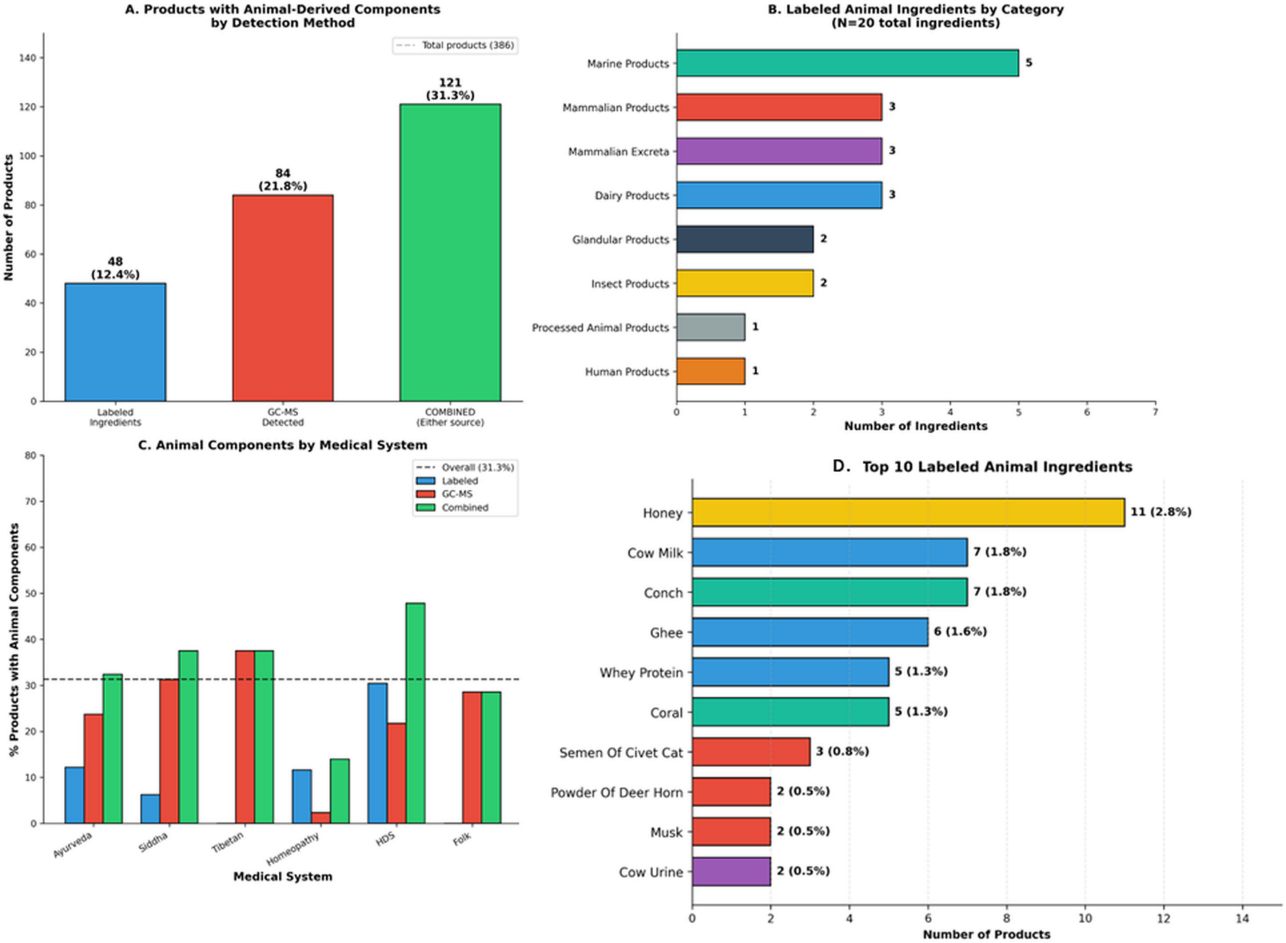
Animal-derived components in CAM products. **(A)** Detection of animal-derived components by methodology. Labeled ingredients identified animal content in 48 products (12.4%), while GC-MS analysis detected animal-derived compounds in 84 products (21.8%). Combined analysis revealed animal-derived content in 121 products (31.3%), indicating substantial undisclosed animal content. Dashed line indicates total products analyzed (N = 386). **(B)** Classification of 20 unique labeled animal-derived ingredients by biological source category: Marine Products (n=5 ingredients), Mammalian Products (n=3), Mammalian Excreta (n=3), Dairy Products (n=3), Glandular Products (n=2), Insect Products (n=2), Processed Animal Products (n=1), and Human Products (n=1). **(C)** Percentage of products containing animal-derived components by medical system, comparing labeled ingredients (blue), GC-MS detected (red), and combined sources (orange). Tibetan medicine and HDS showed the highest combined rates; Homeopathy showed the greatest discrepancy between labeled and detected content. Dashed line indicates overall prevalence (31.3%). **(D)** Top 10 labeled animal-derived ingredients by product frequency: Honey (11 products, 2.8%), Cow Milk (7, 1.8%), Conch (7, 1.8%), Ghee (6, 1.6%), Whey Protein (5, 1.3%), Coral (5, 1.3%), Civet Cat Secretion (3, 0.8%), Deer Horn (2, 0.5%), Musk (2, 0.5%), and Cow Urine (2, 0.5%).

Analysis of animal-derived content employed dual methodology: systematic review of labeled ingredients and gas chromatography-mass spectrometry (GC-MS) detection of animal-specific biomarkers. GC-MS detection of animal-derived biomarkers confirms the presence of animal-origin compounds but cannot distinguish between intentional formulation ingredients and incidental contamination during manufacturing or processing. The term ‘undisclosed’ refers to the absence of label declaration, irrespective of the mechanism of introduction. From labeled ingredients alone, 48 products (12.4%) contained 20 unique animal-derived components spanning marine products (conch, coral, pearl), dairy derivatives (cow milk, ghee, whey protein), mammalian secretions (civet semen, deer horn), mammalian excreta (cow urine, goat urine, elephant excreta), insect products (honey), glandular extracts (pepsinum from pig stomach, thyroidinum from cow or sheep), and human-derived substances (breast milk). GC-MS analysis substantially expanded detection, identifying definitive animal markers in 84 products (21.8%), including cholesterol (25 products, 6.5%), vaccenic acid – a specific ruminant biomarker (12 products, 3.1%), and ursodeoxycholic acid (4 products, 1%). Combined analysis revealed that 121 products (31.3%) contained animal-derived components, with GC-MS detecting nearly twice the prevalence identified through labeling alone – indicating significant undisclosed animal content (**Figure 6**).

#### Labeled and detected heavy metal content and clinical associations

**Figure 7:**
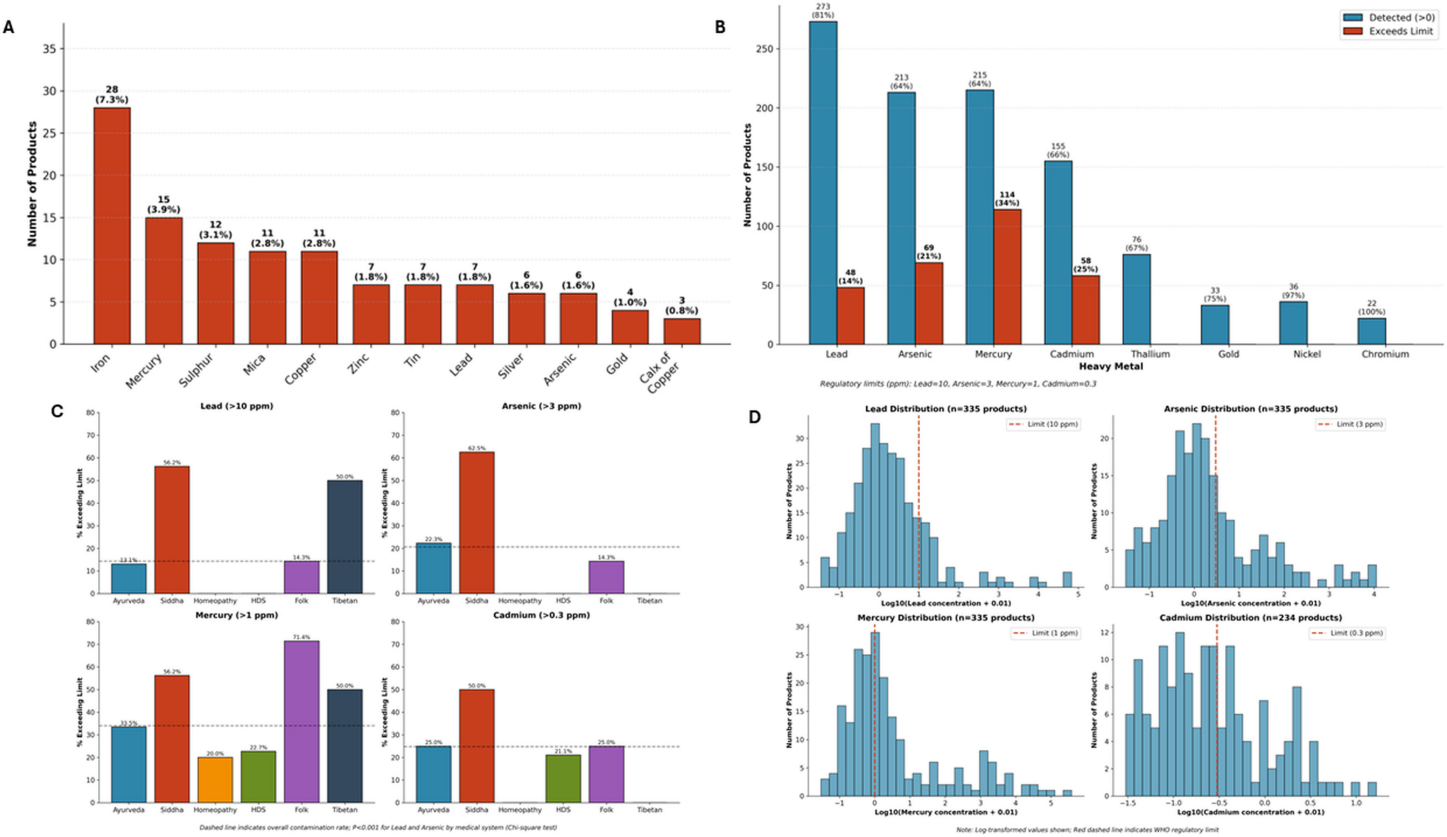
Heavy metal content in CAM products. **(A)** Distribution of labeled heavy metal ingredients across 386 products. Iron was most prevalent (n=28, 7.3%), followed by Mercury (n=15, 3.9%), Sulphur (n=12, 3.1%), Mica (n=11, 2.8%), Copper (n=11, 2.8%), and others including Zinc, Tin, Lead, Silver, Arsenic, Gold, and Calx of Copper. **(B)** Quantitative heavy metal detection (n=335 products tested) showing products with detectable levels (blue) versus products exceeding WHO/regulatory limits (red). Detection rates: Lead 81% (273/335), Arsenic 64% (213/335), Mercury 64% (215/335), Cadmium 66% (155/234). Exceedance rates: Mercury 34% (114 products >1 ppm), Cadmium 25% (58 products >0.3 ppm), Arsenic 21% (69 products >3 ppm), Lead 14% (48 products >10 ppm). **(C)** Heavy metal exceedance rates by traditional medical system. Siddha products demonstrated significantly higher contamination rates for Lead (56.2%) and Arsenic (62.5%) compared to other systems (P<0.001 for both). Folk medicine showed the highest Mercury exceedance (71.4%). Dashed lines indicate overall contamination rates. **(D)** Log-transformed concentration distributions for Lead, Arsenic, Mercury, and Cadmium across tested products. Red dashed vertical lines indicate WHO regulatory limits. Note the substantial proportion of products with concentrations exceeding safety thresholds.

Heavy metal quantification was performed on 335 of 386 products (86.8%); cadmium analysis was available for 234 products (60.6%). The remaining products could not be tested due to insufficient sample quantity. All product-level percentages in this section refer to the number of products tested for the respective metal, not the total of 386 products. Heavy metal analysis employed dual methodology: documentation of labeled metallic ingredients and quantitative detection using ICP-AES across 14 metals. Labeled ingredients (**Figure 7A**) disclosed heavy metals in only 38.5% of patients (35/91), predominantly iron (28 products, 7.3%) and mercury (15 products, 3.9%). However, analytical testing revealed (**Figure 7B**) substantially greater contamination: among 335 products tested, detection rates were 81.5% for lead, 66.2% for cadmium, 64.2% for mercury, and 63.6% for arsenic, with WHO permissible limits exceeded in 34.0% (mercury >1 ppm), 24.8% (cadmium >0.3 ppm), 20.6% (arsenic >3 ppm), and 14.3% (lead >10 ppm) of products. Extreme concentrations indicating intentional metal addition consistent with Ayurvedic *bhasma* (metallo-mineral) preparations were observed, with maximum values of 383,663 ppm for mercury, 67,616 ppm for lead, and 11,262 ppm for arsenic. Contamination rates varied significantly by medical system (**Figures 7C, D**): Siddha products demonstrated the highest prevalence of lead (56.2%) and arsenic (62.5%) exceedances compared to Ayurveda (13.1% and 22.3%, respectively; P<0.001 for both), while regulated and unregulated products showed similar contamination rates. At the patient level, 67% (61/91) were exposed to at least one heavy metal exceeding WHO limits.

**Figure 8:**
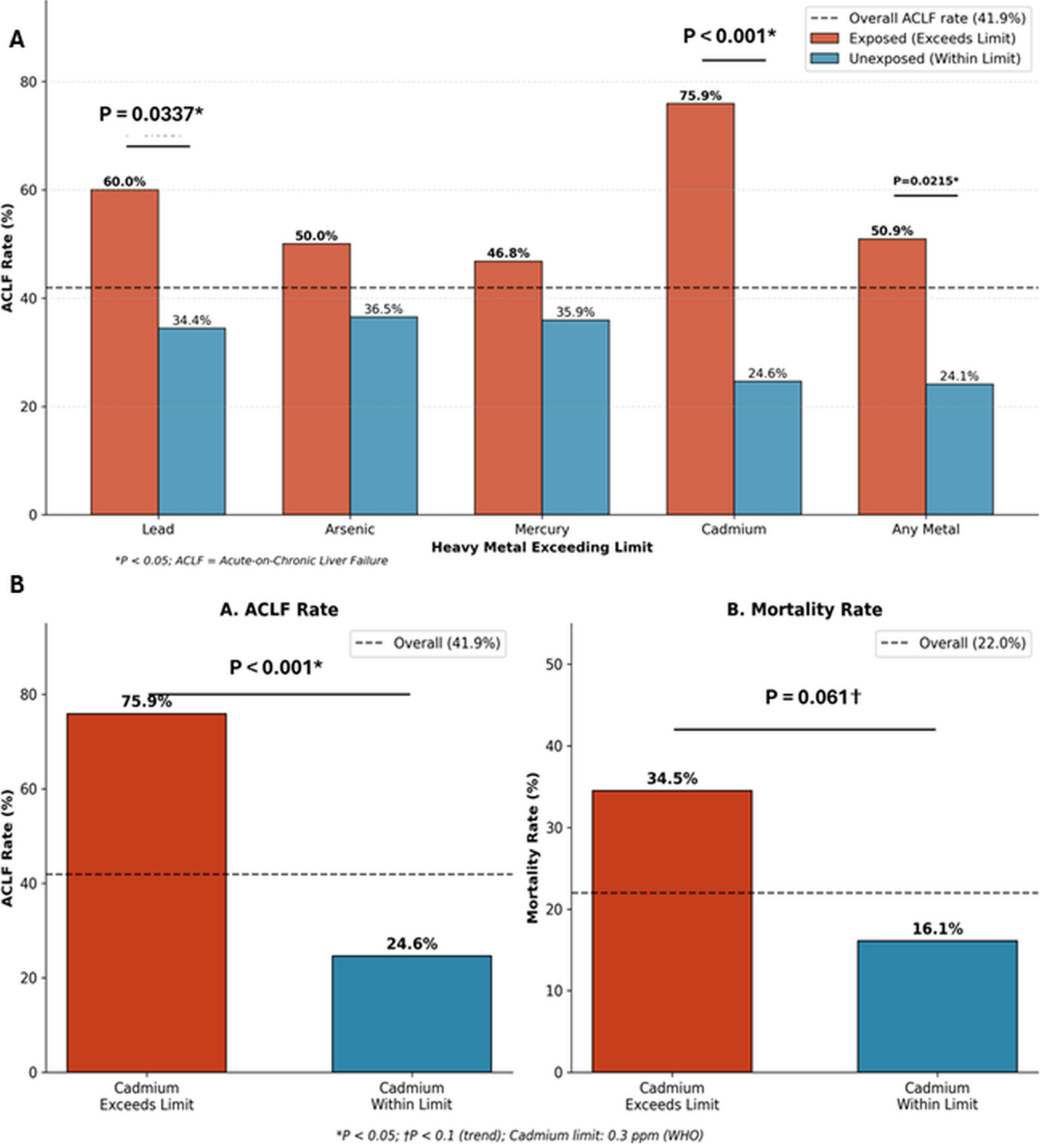
Heavy metal exposure and clinical outcomes. **(A)** ACLF rates stratified by heavy metal exposure exceeding WHO limits. Cadmium exposure showed the strongest association with ACLF (75.9% vs 24.6%, P<0.001), followed by Lead (60.0% vs 34.4%, P = 0.034) and any heavy metal exposure (50.9% vs 24.1%, P = 0.022). Arsenic and Mercury showed elevated but non-significant ACLF rates in exposed patients. Red bars indicate patients with exposure exceeding limits; blue bars indicate unexposed patients. Dashed line represents overall ACLF rate (41.9%). **(B)** Detailed analysis of cadmium exposure and clinical outcomes. Left panel: Patients with cadmium exceeding WHO limits (>0.3 ppm) had dramatically higher ACLF rates compared to unexposed patients (75.9% vs 22.6%, P<0.0001, FDR q<0.0001). Right panel: Cadmium exposure showed elevated but not statistically significant mortality after FDR correction (34.5% vs 16.1%, uncorrected P = 0.061, FDR q=0.24). Dashed lines indicate overall ACLF rate (41.9%) and mortality rate (22.0%).

At the patient level, heavy metal exposure exceeding WHO limits was common: mercury >1 ppm in 51/91 patients (56.0%), arsenic >3 ppm in 38/91 patients (41.8%), lead >10 ppm in 29/91 patients (31.9%), and cadmium >0.3 ppm in 29/91 patients (31.9%). Overall, 61/91 patients (67.0%) were exposed to at least one heavy metal exceeding WHO limits. Cadmium emerged as the most significant predictor of adverse outcomes, with exposed patients demonstrating dramatically higher ACLF rates (75.9% vs 22.6%, P<0.0001, FDR q<0.0001). The association with mortality did not reach statistical significance after correction for multiple comparisons (34.5% vs 16.1%, uncorrected P = 0.061, FDR q=0.24) (**Figures 8A, B**). Lead exposure was also significantly associated with ACLF (60.0% vs 34.4%, P = 0.034), and any heavy metal exceedance increased ACLF risk (50.9% vs 24.1%, P = 0.022). Notably, while heavy metal exposure strongly predicted ACLF development, it was not significantly associated with mortality.

#### GC-MS analysis of CAM content and clinical associations

**Figure 9:**
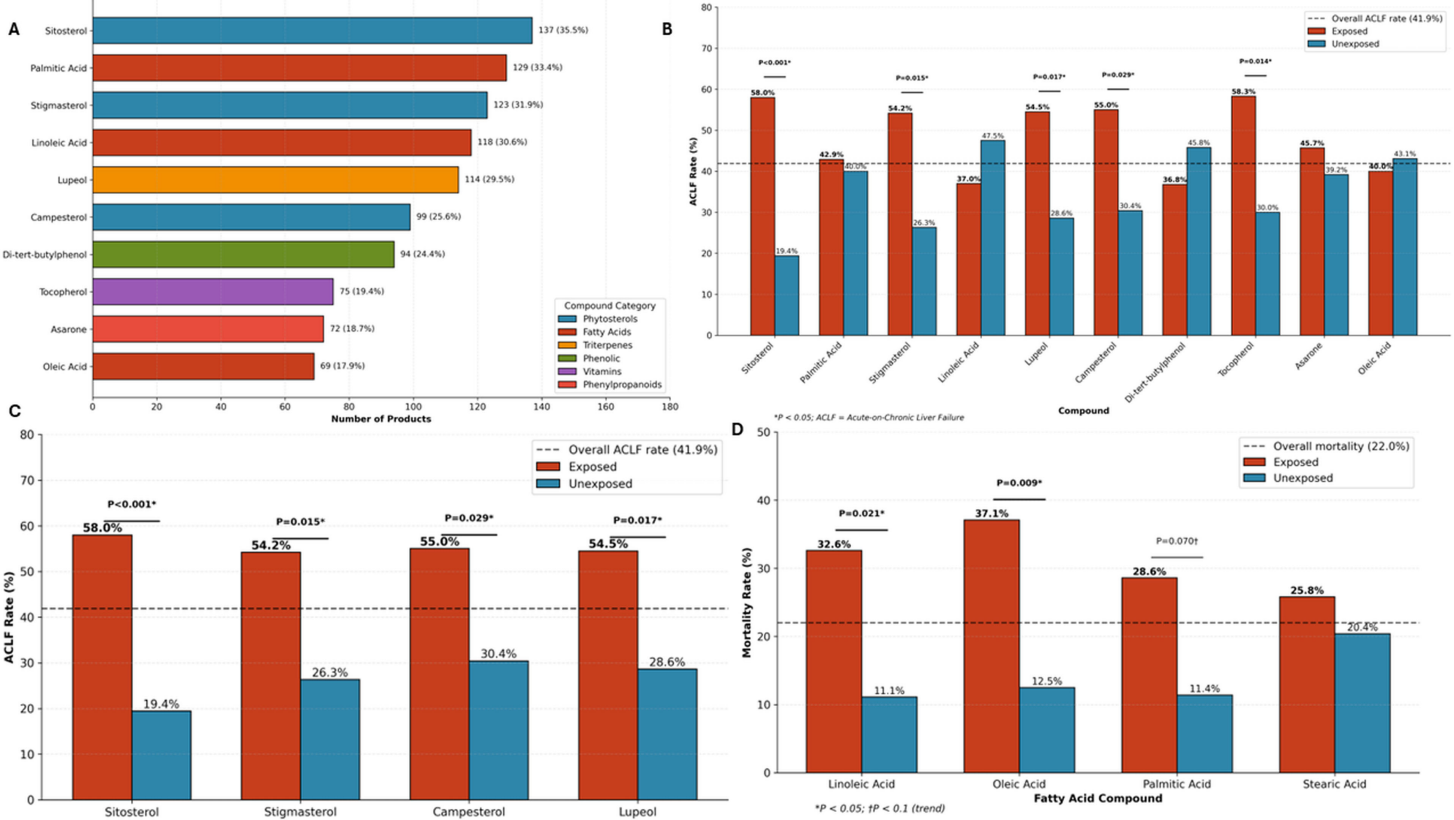
GC-MS compound profiling and clinical associations. **(A)** Top 10 most frequently detected compounds by GC-MS analysis across 386 products, color-coded by phytochemical category: Phytosterols (gray) including Sitosterol (137 products, 35.5%), Stigmasterol (123, 31.9%), and Campesterol (99, 25.6%); Fatty Acids (red) including Palmitic Acid (129, 33.4%), Linoleic Acid (118, 30.6%), and Oleic Acid (69, 17.9%); Triterpenes (orange) including Lupeol (114, 29.5%); Phenolics (green) including Di-tert-butylphenol (94, 24.4%); Vitamins (purple) including Tocopherol (75, 19.4%); and Phenylpropanoids (pink) including Asarone (72, 18.7%). **(B)** ACLF rates by compound exposure for top 10 compounds. Sitosterol, Stigmasterol, Lupeol, Campesterol, and Tocopherol showed significantly elevated ACLF rates in exposed patients. Dashed line indicates overall ACLF rate (41.9%). **(C)** Significant phytosterol and triterpene associations with ACLF: Sitosterol (58.0% vs 19.4%, P<0.001), Stigmasterol (54.2% vs 26.3%, P = 0.015), Campesterol (55.0% vs 30.4%, P = 0.029), and Lupeol (54.5% vs 28.6%, P = 0.017). **(D)** Fatty acid associations with mortality: Oleic Acid (37.1% vs 12.5%, P = 0.009), Linoleic Acid (32.6% vs 11.1%, P = 0.021), Palmitic Acid (28.6% vs 11.4%, P = 0.070, trend). Dashed line indicates overall mortality rate (22.0%). *P<0.05; †P<0.1 (trend). After FDR correction across 226 compounds, 29 compounds remained significantly associated with ACLF (q<0.05), while no compound-mortality associations survived correction (all q>0.34).

Gas chromatography-mass spectrometry (GC-MS) analysis identified 289 unique compounds across 356 products (92.2%), with a mean of 13.1 ± 10.6 compounds per product. Compounds were classified into established phytochemical categories, with sesquiterpenes (29 compounds), fatty acids (18 compounds), and phytosterols (4 compounds) representing the most prevalent classes. The most frequently detected compounds were sitosterol (137 products, 35.5%), palmitic acid (129, 33.4%), stigmasterol (123, 31.9%), linoleic acid (118, 30.6%), and lupeol (114, 29.5%) (**Figure 9A**). Clinical outcome analysis revealed striking associations between specific compound classes and ACLF development (**Figures 9B-D**). Phytosterols demonstrated the strongest associations: sitosterol exposure was associated with an ACLF rate of 58.0% versus 19.4% in unexposed patients (P<0.001, FDR q=0.016), with stigmasterol (54.2% vs 26.3%, P = 0.015, FDR q=0.037) and campesterol (55.0% vs 30.4%, P = 0.029, FDR q=0.049) showing similar patterns. These associations remained statistically significant after FDR correction. The triterpene lupeol was also significantly associated with ACLF (54.5% vs 28.6%, P = 0.017). Conversely, fatty acids showed associations with mortality in uncorrected analyses: oleic acid exposure was associated with mortality of 37.1% versus 12.5% in unexposed patients (uncorrected P = 0.009, FDR q=0.34), and linoleic acid with 32.6% versus 11.1% (uncorrected P = 0.021, FDR q=0.34). However, neither association remained statistically significant after FDR correction across 226 tested compounds, indicating these findings should be considered exploratory – also potentially reflecting concentrated lipid extracts with hepatotoxic potential distinct from dietary sources. Paradoxically, tocopherol (vitamin E) was associated with increased ACLF (58.3% vs 30.0%, P = 0.014), likely representing confounding by co-occurrence with other harmful constituents rather than direct toxicity. After applying Benjamini-Hochberg FDR correction across 226 tested compounds, 29 compounds remained significantly associated with ACLF (q<0.05), including sitosterol (q=0.016), piperine (q=0.016), squalene (q=0.016), and stigmasterol (q=0.037). Notably, no compound associations with mortality remained statistically significant after FDR correction (all q>0.34), indicating that the observed mortality associations should be considered exploratory and hypothesis-generating rather than confirmatory. These findings suggest that ubiquitous phytochemicals, when present in concentrated botanical preparations, may contribute to hepatotoxicity through mechanisms distinct from their recognized physiological roles.

#### Pharmaceutical drugs adulteration of CAM and clinical associations

**Figure 10:**
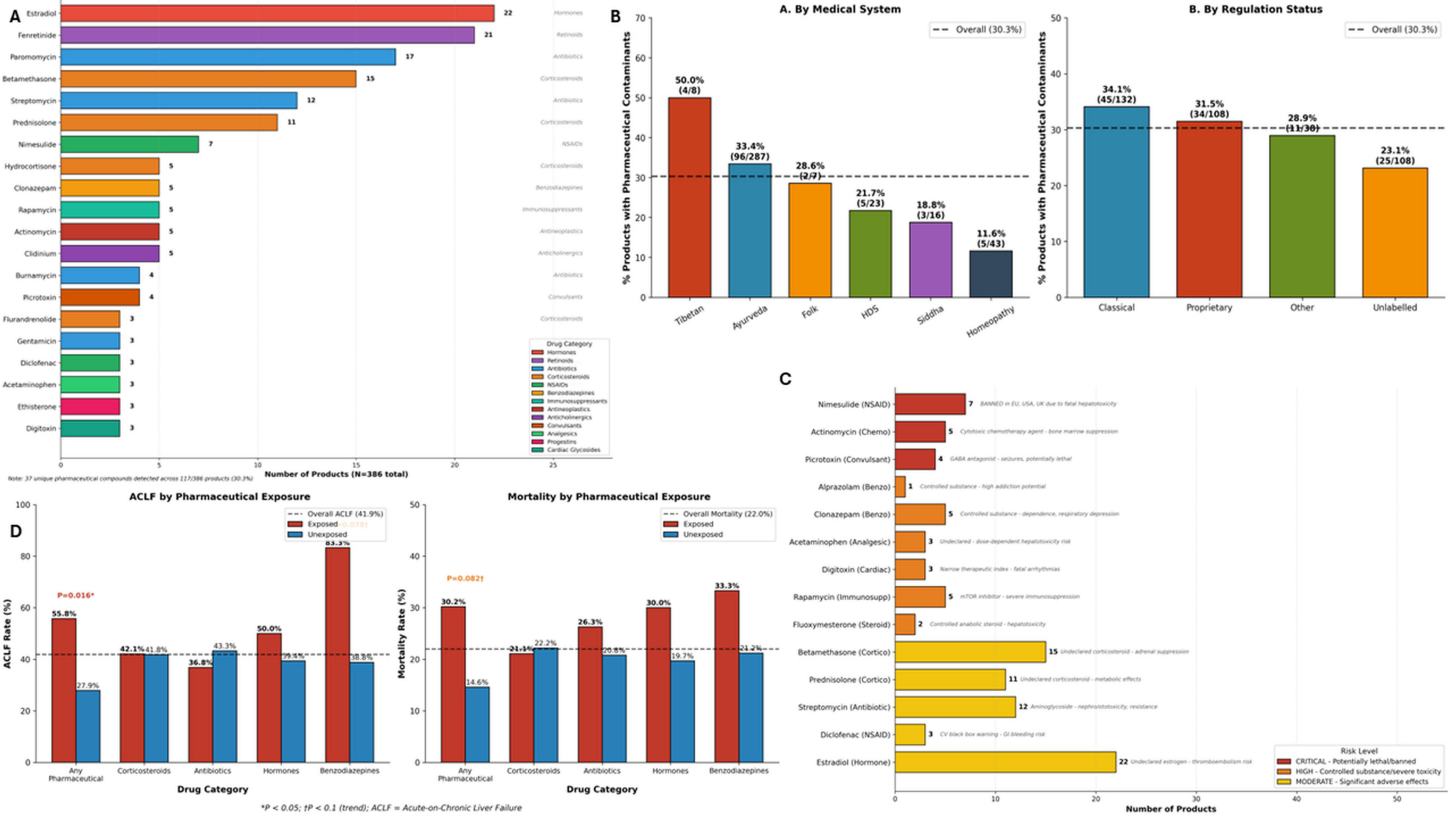
Pharmaceutical contamination in CAM products. **(A)** Frequency distribution of 37 pharmaceutical contaminants detected across 117 products (30.3%), color-coded by drug category. Most prevalent: Estradiol (22 products), Fenretinide (21), Paromomycin (17), Betamethasone (15), Streptomycin (12), Prednisolone (11), and Nimesulide (7). Categories include Hormones, Retinoids, Antibiotics, Corticosteroids, NSAIDs, Benzodiazepines, Immunosuppressants, Antineoplastics, Anticholinergics, Analgesics, Progestins, Convulsant, and Cardiac Glycosides. **(B)** Pharmaceutical contamination rates by medical system (left) and regulatory status (right). Tibetan medicine showed the highest rate (50.0%), followed by Ayurveda (33.4%). Paradoxically, Classical regulated products showed higher contamination (34.1%) than Unlabeled products (23.1%). Dashed lines indicate overall contamination rate (30.3%). **(C)** High-risk pharmaceutical contaminants categorized by safety concern: CRITICAL (red) = potentially lethal/banned drugs including Nimesulide (banned for hepatotoxicity), Actinomycin (cytotoxic chemotherapy), and Picrotoxin (convulsant); HIGH (orange) = controlled substances including benzodiazepines and anabolic steroids; MODERATE (yellow) = drugs with significant adverse effects including undeclared corticosteroids, antibiotics, and hepatotoxic analgesics. **(D)** Clinical outcomes by pharmaceutical exposure category. Left: Any pharmaceutical exposure was significantly associated with ACLF (55.8% vs 27.9%, P = 0.016), with benzodiazepine exposure showing the highest ACLF rate (83.3%). Right: Pharmaceutical exposure showed a trend toward increased mortality (30.2% vs 14.6%, P = 0.082). Dashed lines indicate overall ACLF (41.9%) and mortality (22.0%) rates. *P<0.05; †P<0.1 (trend).

GC-MS analysis revealed undeclared pharmaceutical contamination in 107 of 386 CAM products (27.7%), with 42 of 91 patients (46.2%) exposed to at least one pharmaceutical adulterant. Analysis identified 37 unique pharmaceutical compounds spanning nine drug categories. The most frequently detected drugs (**Figure 10A**) were estradiol (22 products, 5.7%), fenretinide (21, 5.4%), paromomycin (17, 4.4%), betamethasone (15, 3.9%), and streptomycin (12, 3.1%). Contamination was observed across all medical systems (**Figure 10B**), with highest rates in Tibetan medicine (50.0%), Ayurveda (33.4%), and folk medicine (28.6%). Paradoxically, classical regulated formulations demonstrated the highest contamination rate (34.1%) compared to unlabeled products (23.1%), suggesting that regulatory classification does not ensure pharmaceutical purity. The spectrum (**Figure 10C**) of contaminants included corticosteroids (betamethasone, prednisolone, hydrocortisone, dexamethasone; 37 products total), antibiotics (paromomycin, streptomycin, gentamicin, amoxicillin; 38 products), NSAIDs (nimesulide, diclofenac, aspirin; 11 products), benzodiazepines (clonazepam, nordazepam, alprazolam; 8 products), hormones (estradiol, ethisterone, medroxyprogesterone; 26 products), and antineoplastic agents (actinomycin; 5 products). Critical safety concerns included detection of nimesulide (7 products) – banned in the European Union, United States, and United Kingdom due to fatal hepatotoxicity – acetaminophen (3 products) and diclofenac (3 products) with established hepatotoxic potential administered to patients with pre-existing liver disease, picrotoxin (4 products) – a potentially lethal GABA antagonist convulsant – and Schedule IV controlled benzodiazepines (8 products) and Schedule III anabolic steroids (fluoxymesterone; 2 products) without patient knowledge or consent.

Pharmaceutical contamination was significantly associated with adverse clinical outcomes (**Figure 10D**) – exposed patients demonstrated higher ACLF rates (55.8% vs 27.9%, P = 0.016, FDR q=0.064) and a trend toward increased mortality (30.2% vs 14.6%, uncorrected P = 0.082). The ACLF association showed borderline significance after FDR correction, with benzodiazepine exposure showing particularly elevated ACLF rates (83.3% vs 38.8%, P = 0.078). These findings underscore a profound failure of quality control at one or more points across the traditional medicine supply chain, potentially including manufacturing, distribution, storage, or point-of-sale handling – with patients unknowingly exposed to prescription medications, controlled substances, and drugs with established hepatotoxicity contraindicated in liver disease.

## Discussion

In our single-center cohort of 91 patients with CAM-related liver injury, ACLF was the commonest and most lethal phenotype (39.6% with 38.9% mortality), driving an overall mortality of 22%. These clinical outcomes occurred in the setting of striking product-level safety failures across 386 implicated preparations which included: widespread heavy-metal contamination with frequent exceedance of accepted limits and extreme concentrations consistent with intentional metallo-mineral addition, substantial pharmaceutical adulteration (30.3%) including corticosteroids, NSAIDs and other prescription agents, frequent undisclosed animal-derived content (31.3%), and high prevalence of botanicals with known or suspected hepatotoxic potential (≥35% of products; >50% of patients exposed to “well-documented” hepatotoxins). Importantly, the strongest signal for mortality was not a specific medical “system” but exposure to unlabeled/untraceable products with a dose-response relationship. These observations align with (and, in several respects, exceed) the broader international experience that herbal products and dietary supplements contribute significantly towards DILI burden and can produce severe outcomes – prompting concern, structured evaluation and regulatory improvements.

The predominance of ACLF (~40%) and its ~5-fold association with death in this cohort underscores a central clinical reality in South/Southeast Asia: CAM exposure often occurs on the background of chronic liver disease and can act as an acute precipitant that rapidly narrows the therapeutic window. This is consistent with Asia-Pacific consensus guidance emphasizing the region’s high DILI burden in part due to ubiquitous traditional medicine use, and with AARC data demonstrating that drug-induced ACLF carries high short-term mortality and that CAM constitutes a large fraction of drug-triggered ACLF events in Asian cohorts (5, 6). Compared with many Western DILI registries, where most HDS-related cases present as acute hepatitis patterns and ACLF is less dominant, our cohort most likely reflects referral bias (tertiary hepatology center), high baseline pre-existing liver disease prevalence in the community, multi-product exposure, and delayed recognition/continued ingestion due to perceived “natural safety.” These factors plausibly explain why mortality in CAM-associated liver injury can be markedly higher in populations with underlying liver disease than in general DILI cohorts described in the West (16).

A particularly actionable finding is the association between unlabeled product exposure and mortality with an apparent dose–response relationship. This is not merely a “documentation” issue: lack of batch details, ingredient lists, or manufacturer identity makes risk assessment, patient counselling, and public-health action almost impossible. The association likely represents a composite of (i) higher probability of dangerous adulteration/contamination, (ii) extreme batch-to-batch variability, and (iii) delayed discontinuation because neither patients nor clinicians can identify what was taken. This aligns with a broader pharmacovigilance message emerging from DILI networks: product characterization improves causality confidence and can materially change attribution in a substantial proportion of cases, supporting the clinical value of retrieving and analyzing implicated products rather than relying on label claims alone (15). Our data extend this concept: when products are unlabeled, the “information deficit” itself becomes a prognostic marker, because it correlates with exposure to an unregulated supply chain.

Heavy-metal contamination was ubiquitous, with high detection rates for lead, cadmium, mercury and arsenic, frequent exceedance of commonly referenced limits, and extreme maximum concentrations suggesting intentional addition in some products. These findings are directionally consistent with long-standing concerns about heavy metals in Ayurvedic and other traditional medicines, including surveys demonstrating measurable lead/mercury/arsenic in a meaningful fraction of products sold internationally (17). Contemporary reviews and risk assessments continue to highlight heavy metals as a recurrent contaminant class in herbal medicines globally. An important distinction exists between intentionally added metal ingredients (as in traditional bhasma preparations where metals are processed according to Ayurvedic pharmaceutical principles) and undisclosed contamination from environmental sources, manufacturing equipment, or ingredient substitution. The WHO limits applied in this study (lead ≤10 ppm, arsenic ≤3 ppm, mercury ≤1 ppm, cadmium ≤0.3 ppm) were established for finished herbal products and may not be directly applicable to metal-containing bhasma formulations designed for therapeutic metal delivery. However, even for such products, the concentrations detected (particularly mercury up to 383,663 ppm and lead up to 67,616 ppm in some products) substantially exceed any traditional dosing rationale and raise significant safety concerns regardless of intentionality. Future studies should separately analyze products with declared versus undisclosed metal content to better characterize risk profiles.

The most notable clinical association in this cohort is cadmium exposure predicting ACLF. While cadmium exposure showed a trend toward higher mortality in uncorrected analysis (P = 0.061), this association did not remain statistically significant after FDR correction (q=0.24), suggesting that cadmium’s primary impact may be on disease severity rather than ultimate survival. This is biologically plausible. Cadmium is a potent toxicant that promotes oxidative stress, mitochondrial dysfunction, disordered lipid metabolism, and inflammatory signaling mechanisms that can precipitate hepatocyte injury and worsen systemic inflammation (18). Epidemiologic syntheses also support an association between cadmium exposure and increased risk of liver disease at the population level (19). In patients with cirrhosis or advanced fibrosis, even modest additional hepatotoxic stressors can plausibly tip the balance toward ACLF through amplified inflammatory responses and reduced hepatic reserve – providing a rational framework for why cadmium might associate more strongly with severity (ACLF) than with mortality per se, where supportive care timing, infections, and organ failures also dominate. A practical implication is that “heavy metals” should not be treated as a theoretical risk: when CAM exposure is suspected in severe presentations, clinicians should consider early toxicology testing and involve poison/toxicology services, consistent with guideline emphasis on systematic evaluation of suspected DILI/herb-induced liver injury (HILI) (20). Additionally, cadmium is a well-established nephrotoxin, and the hepatorenal axis may contribute to adverse outcomes in patients with concurrent kidney injury. Cadmium accumulates primarily in the kidney, where it causes proximal tubular dysfunction and can lead to chronic kidney disease with prolonged exposure. In patients with underlying liver disease, concurrent renal impairment represents a particularly poor prognostic combination. Although renal function data were not systematically collected in this cohort, future studies should assess both hepatic and renal outcomes in patients with CAM-associated cadmium exposure.

The detection of undeclared pharmaceuticals in 30.3% of implicated products is a blunt indicator of intentional or negligent adulteration. Importantly, adulteration was not confined to unlabeled products; classical “regulated” formulations also showed high contamination. This mirrors prior multi-modal audits of traditional medicines where substantial proportions contained undeclared pharmaceuticals, heavy metals, and substituted biological materials – sometimes including controlled or high-risk drugs (21, 22). It also aligns with analyses of seized traditional products in which steroids and NSAIDs appear as common adulterants, consistent with “rapid symptom relief” incentives (23). Clinically, our observation that pharmaceutical adulteration associates with higher ACLF rates and a trend toward higher mortality is credible because several detected drug classes have recognized hepatotoxic potential and can be catastrophic when unknowingly administered to patients with established liver disease (e.g., interacting sedatives, steroids, or nephrotoxic agents contributing to multi-organ failure). Undisclosed inclusion of prescription-only drugs in CAM products eliminates informed consent, creates interaction risk, and undermines clinical attribution when adverse events occur.

A large fraction of CAM products contained botanicals with documented or suspected hepatotoxicity, and most patients were exposed to at least one such ingredient. This is consistent with the evolving global literature on HILI, where certain agents (including ashwagandha, turmeric/curcumin, green tea extract, *Tinospora cordifolia*, and others) recur across case series and registries (24). Our study observed non-significant trend-level associations between *Tinospora cordifolia* and *Plumbago zeylanica* exposure and ACLF development after FDR correction (q=0.50 for both). These findings were underpowered for definitive ingredient-level causal claims. *Tinospora* has been linked, in published literature, to immune-mediated liver injury phenotypes in susceptible individuals, and *Plumbago* contains plumbagin, a reactive quinone with plausible hepatotoxic mechanisms. In this setting, “novelty” likely reflects the reality that the clinical event is rarely attributable to a single named herb: it is the interaction of host susceptibility (pre-existing liver disease, metabolic disorders, immune predisposition), cumulative botanical toxins, and hidden contaminants that determines phenotype.

The identification of animal-derived markers in nearly one-third of products, with GC-MS doubling detection compared with labels, is consistent with prior investigations showing undeclared biological materials, including animal taxa, in traditional medicines (21, 22). While animal content is not automatically hepatotoxic, it matters for (i) informed consent and religious/vegetarian restrictions, (ii) zoonotic contamination risk, and (iii) endangered species protection. The fact that under-labeling is common also supports a broader inference: if manufacturers/practitioners omit animal ingredients, they may also omit (or be unaware of) other harmful constituents – again reinforcing traceability as a safety determinant.

Our study showed novel associations between phytosterols (e.g., sitosterol, stigmasterol) and ACLF that remained statistically significant after FDR correction. In contrast, the associations between certain fatty acids (oleic/linoleic) and mortality observed in uncorrected analyses did not survive multiple testing correction and should be considered hypothesis-generating rather than confirmatory. These are intriguing because these compounds are ubiquitous in plants and foods. However, two rational interpretations deserve emphasis: (i) these molecules may have been proxies for particular preparation types such as oil-rich extracts, resins, concentrated lipid matrices, or specific botanical clusters, rather than the direct toxicity drivers. In multi-ingredient products, frequent co-occurrence can generate strong statistical associations even if the causal agent is another correlated constituent; and (ii) phytosterols have been implicated experimentally and clinically in cholestasis/liver injury in specific contexts (notably parenteral nutrition–associated liver disease), suggesting that high exposure states or impaired bile handling could make sterol-rich formulations biologically relevant (25). Specifically, stigmasterol, which was also found to be associated with severe liver disease phenotype in our study, has been shown to promote liver injury in pre-clinical studies using plant-based (soy) lipid emulsions (26). For fatty acids, hepatotoxicity is not typical for dietary intake, but concentrated fatty-acid supplements (e.g., conjugated linoleic acid formulations) have been reported in association with acute hepatitis and even acute liver failure, supporting biological plausibility for “non-food” exposure patterns (27). Additionally, these ubiquitous plant compounds may serve as markers for specific product types (particularly oil-based preparations) rather than representing direct hepatotoxic agents. The co-occurrence of phytosterols with other bioactive compounds in complex botanical matrices precludes attribution of adverse effects to any single constituent. These signals should therefore be framed as hypothesis-generating: they point toward preparation chemistry (concentration, solvent extraction, lipid matrices) as an underappreciated determinant of severity, and they justify follow-up work with quantitative assays, dose estimation, and clustering/mixture modeling.

### Limitations, and implications

This study has several limitations that merit consideration. We would like to highlight the fact that critically, no validated causality assessment instrument exists for multi-product HILI scenarios. RUCAM and similar tools were designed for single-drug exposures and perform unreliably when patients consume multiple products with unknown or adulterated composition. Our approach – temporal association after exclusion of competing etiologies, combined with expert hepatologist adjudication – represents the pragmatic standard used by major DILI networks (DILIN, LATINDILI) for complex cases, but lacks the formal reproducibility of structured instruments. Individual-patient causality cannot be established with confidence in this setting, and the associations reported should be interpreted as statistical correlations observed in a cohort of patients whose clinical presentations were temporally associated with CAM exposure.

First, the retrospective observational design at a single tertiary hepatology referral center introduces both selection and referral bias, as our cohort likely over-represents severe cases and those with pre-existing liver disease compared to the broader population of CAM users experiencing adverse events. Second, the inclusion criterion requiring retrievable product samples may have excluded patients with less documented exposures, potentially underestimating the true burden of CAM-related hepatotoxicity. Third, the prevalent multi-product exposure pattern with correlated ingredients limits causal attribution to individual botanicals, metals, or adulterants; observed associations should therefore be interpreted as hypothesis-generating rather than definitively causal. Fourth, GC-MS analysis was primarily qualitative (presence/absence) rather than quantitative, precluding dose-response characterization for detected compounds. Fifth, the absence of a matched control group of CAM users without adverse events precludes estimation of population-attributable risk for specific contaminants or ingredients. Sixth, the GC-MS analysis was primarily qualitative, precluding dose-response characterization of compound-outcome relationships. The minimum match threshold utilized provides reasonable confidence in compound identification but cannot exclude occasional misidentification, particularly for structurally similar compounds, metabolites, or degradation products. Confirmatory analysis using orthogonal methods (LC-MS/MS with reference standards) was not performed and represents a limitation of this study. The duration of CAM product use prior to presentation was not systematically documented in our cohort, precluding dose-duration response analysis (even though CAM related liver injury is dose-independent). Additionally, interviewer and recall bias in CAM exposure history may have affected exposure classification, as patients or family members may have incompletely reported or recalled CAM use patterns. Importantly, the reliance on GC-MS/MS as the sole chromatographic platform means that non-volatile, thermally labile, highly polar, or high molecular weight compounds (>~600 Da) would not be detected. This includes clinically relevant hepatotoxic compounds such as pyrrolizidine alkaloids (without prior derivatization), peptide toxins (e.g., amatoxins from Amanita species contamination), large-molecule mycotoxins, and certain polar pharmaceutical adulterants (e.g., metformin, ACE inhibitors, proton pump inhibitors). The true prevalence of both hepatotoxic compound exposure and pharmaceutical adulteration in this cohort may therefore be underestimated. Future studies should employ complementary LC-MS/MS analysis to achieve comprehensive analytical coverage of the full chemical space relevant to traditional medicine safety assessment. Furthermore, no patient-level biological exposure data (blood or urine heavy metal concentrations) were obtained. Product-level concentrations served as surrogate exposure markers, consistent with established pharmacovigilance methodology for traditional medicine safety assessment, but do not directly reflect individual absorbed dose, which depends on formulation bioavailability, duration and frequency of consumption, and individual pharmacokinetics. Finally, findings from this South Indian cohort may not generalize to other geographic regions with different CAM traditions, product supply chains, or underlying disease prevalences. Detailed baseline liver disease characterization and severity scores (Child-Pugh class, MELD score) were not systematically documented in our cohort, precluding stratified analysis by underlying liver disease status. Given that 45.1% of patients presented with ACLF (39.6%) or acute decompensation (5.5%), a substantial proportion had pre-existing chronic liver disease that may have modified individual susceptibility to CAM-induced hepatotoxicity. The associations observed in this study should therefore be interpreted in the context of syndromic presentations and related outcomes, and future prospective studies should incorporate standardized baseline liver disease assessment. Additionally, the requirement for retrievable product samples for laboratory analysis may have introduced selection bias. Patients with more severe illness requiring emergent hospitalization may have been less likely to retain CAM products, while those with better health literacy and recall may have been more likely to provide samples for analysis. The direction of this potential bias is uncertain and could affect either the exposure characterization or outcome associations. This selection criterion, while necessary for our comprehensive analytical approach, limits generalizability to the broader population of CAM users experiencing adverse events. Several additional important caveats apply to the interpretation of our findings. Reverse causation was also possible: patients with more severe underlying liver disease may preferentially seek traditional remedies, leading to spurious associations between CAM exposure and poor outcomes. The cross-sectional nature of exposure assessment precludes establishment of temporal relationships necessary for causal inference. Prospective cohort studies with standardized baseline assessment and longitudinal follow-up would be required to establish causality. Future prospective studies should track patients excluded due to unavailable products to quantify this potential bias.

We acknowledge that existing regulatory frameworks, including the Drugs and Cosmetics Act (1940), AYUSH GMP guidelines, and the establishment of pharmacovigilance programs under the National AYUSH Mission, represent important steps toward traditional medicine safety. Several state-level initiatives have also implemented quality testing of Ayurvedic products, and the Ayurvedic Pharmacopoeia of India provides monograph-level quality standards for classical formulations. However, our findings suggest that implementation gaps persist, particularly regarding awareness and education on lack of objective evidence, the use of potentially toxic botanicals, enforcement of labeling requirements for practitioner-dispensed products, systematic heavy metal surveillance at the batch level, and detection of pharmaceutical adulteration. The proposed recommendations should therefore be viewed as strengthening existing systems rather than replacing them.

To conclude, we demonstrate that CAM-associated liver injury frequently presents as ACLF with substantial mortality occurring against a backdrop of pervasive product safety failures. Heavy metal contamination was near-universal, with cadmium exposure emerging as a significant predictor of ACLF. Undeclared pharmaceutical adulteration affected nearly one-third of products, including hepatotoxic NSAIDs, corticosteroids, and controlled substances, while undisclosed animal-derived content was detected in nearly one-third of products. Critically, consumption of unlabeled products, reflecting an untraceable supply chain, was the strongest predictor of mortality, independent of specific contaminants. These findings indicate that preventable, supply-chain-driven toxicity acting on vulnerable hosts with limited hepatic reserve substantially contributes to poor outcomes. Clinically, structured CAM history-taking, early discontinuation upon suspicion, product retrieval for analysis, and integration of toxicology services should be prioritized in severe presentations. From a regulatory standpoint, mandatory product traceability, batch-level heavy metal and adulterant surveillance, and enforcement of labeling standards represent essential patient safety interventions – because current regulatory classification and labeling alone is demonstrably insufficient to ensure product safety.

## Data Availability

The original contributions presented in the study are included in the article/supplementary material. Further inquiries can be directed to the corresponding author.
